# Does adding intraperitoneal paclitaxel to standard intraperitoneal regimen yield incremental survival? A propensity score-matched cohort study

**DOI:** 10.1186/s40880-016-0105-3

**Published:** 2016-05-09

**Authors:** Yen-Hou Chang, Chien-Hsing Lu, Ming-Shyen Yen, Wai-Hou Lee, Yi Chang, Wei-Pin Chang, Chi-Mu Chuang

**Affiliations:** Institute of Clinical Medicine, School of Medicine, National Yang-Ming University, Taipei, 11221 Taiwan China; Department of Obstetrics and Gynecology, Taichung Veterans General Hospital, Taichung, 40705 Taiwan China; Institute of Biomedical Sciences, National Chung Hsing University, Taichung, 40227 Taiwan China; Rong-Hsing Research Center for Translational Medicine, National Chung Hsing University, Taichung, 40227 Taiwan China; Department of Obstetrics and Gynecology, Taipei Veterans General Hospital, Taipei, 11217 Taiwan China; Department of Healthcare Management, Yuanpei University, Hsinchu, 30015 Taiwan China

**Keywords:** Epithelial ovarian, tubal, and peritoneal cancer, Intraperitoneal chemotherapy, Propensity score, Survival, Clinical trials

## Abstract

We recruited consecutive patients with stage III epithelial ovarian, tubal, and peritoneal cancers who had optimal residual tumor after primary cytoreductive surgery and who received intraperitoneal chemotherapy between 2002 and 2012. Two propensity score-matched sample cohorts were created. We found that the addition of paclitaxel as a second intraperitoneal agent on a 3-week dosing schedule did not yield significant incremental survival benefits over the intraperitoneal delivery of a single cisplatin-based regimen. If our findings could be confirmed by a prospective randomized study, then it would be interesting to explore the efficacy of shifting back to a dose-dense intraperitoneal delivery of paclitaxel or a dose-dense delivery of a new formulation of paclitaxel for the patients with stage III epithelial ovarian, tubal, and peritoneal cancers.

## Background

Currently, the standard intraperitoneal chemotherapy protocol for the treatment of stage III epithelial ovarian, tubal, and peritoneal cancers follows that of the Gynecologic Oncology Group (GOG) 114 trial (namely, intraperitoneal delivery of 100 mg/m^2^ of cisplatin on day 2) [1] or the GOG 172 trial (intraperitoneal delivery of 100 mg/m^2^ of cisplatin on day 2 and intraperitoneal delivery of 60 mg/m^2^ of paclitaxel on day 8) [2]. These protocols share a common intravenous delivery of 135 mg/m^2^ of paclitaxel on day 1. Importantly, the GOG 114 trial called for two cycles of a high dose of intravenous carboplatin (area under the curve, 9) before formal intraperitoneal treatment was administered. Although intraperitoneal chemotherapy has demonstrated superiority in the treatment of ovarian cancer, in clinical practice the completion rate of six assigned intraperitoneal cycles reached only 71% in the GOG 114 trial and 42% in the GOG 172 trial. Catheter-induced complications remain a major problem for patients who are unable to complete the assigned cycles [3].

In the present study, we compared the efficacy between GOG 114 and GOG 172 using survival as the primary end-point.

## Patients and methods

**Fig. 1:**
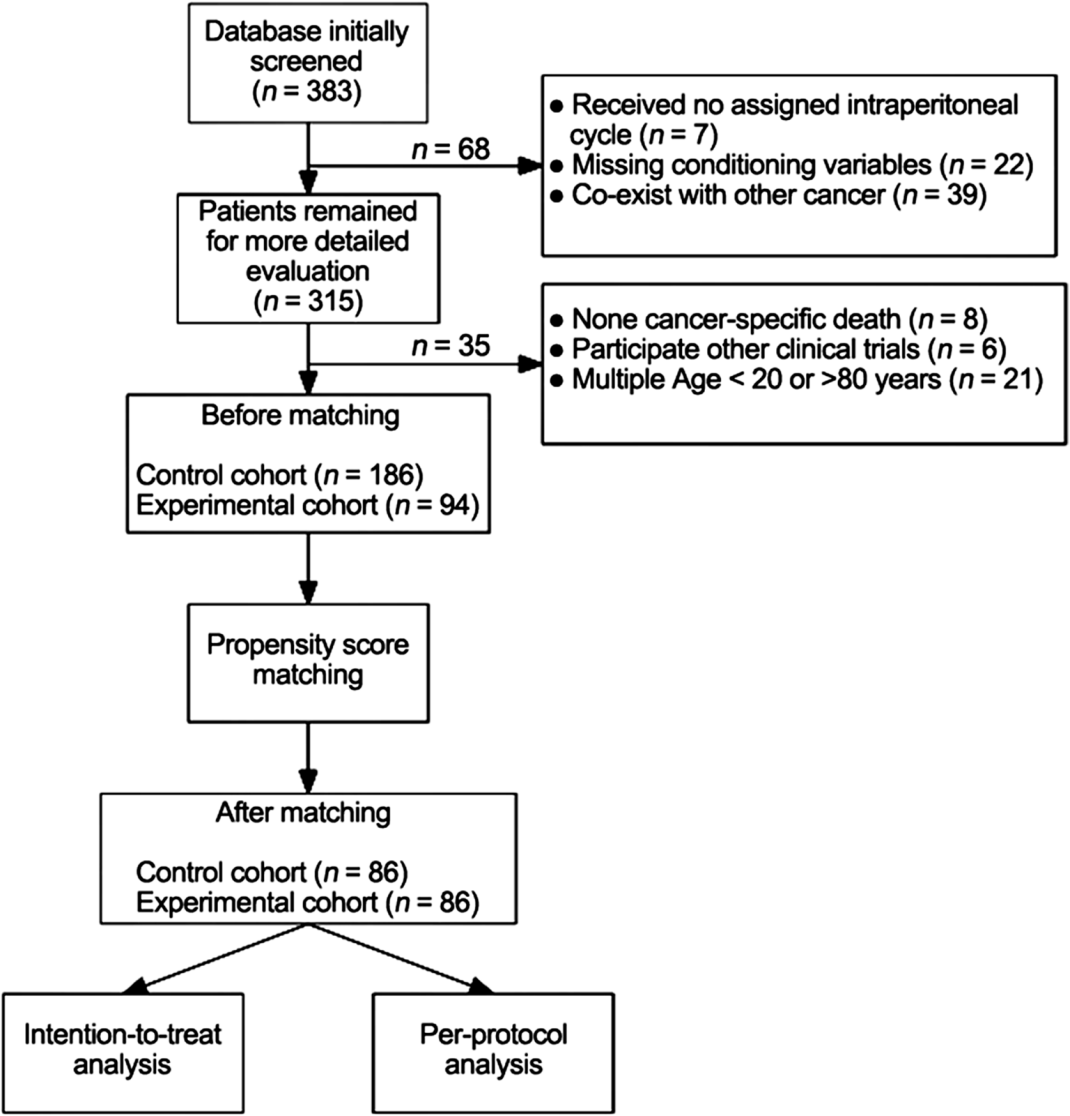
Flow chart of the screening and propensity score-matching process

We collected the clinical data of patients with stage III epithelial ovarian, tubal, and peritoneal cancer between January 2002 and December 2012 who were treated in the Institutional Review Board of Taipei Veterans General Hospital. Patients in the control cohort were treated according to the protocol described in the GOG 114 trial [1]; patients in the experimental cohort were treated according to the protocol described in the GOG 172 trial [2]. A flow chart describing the screening and matching process is shown in Fig. 1.

We used a propensity score-matching technique to compare two intraperitoneally treated cohorts of patients. The technique of propensity score, which is a covariate summary score, was used for the analysis in the present study [4, 5].

The matching procedure involved a two-step analysis. In step 1, we used 10 conditioning variables to develop propensity scores. In step 2, we used an algorithm of the nearest neighbor matching within a specified caliper distance (0.25 σ in the current study, where σ was the standard deviation of logit of propensity score) without replacement to create matched samples [6, 7]. Thus, for a given patient in the experimental group, we identified all of the patients in the control cohort whose propensity scores lay within a specified distance of that of the patient in the experimental group. From this restricted set of control patients, we matched the patient in the control group whose propensity score was closest to that of the patient in the experimental group. Eventually, this created two samples of equal size (1:1 matching).

We analyzed progression-free survival and overall survival in this study. All analyses were performed using STATA SE software, version 12 (Stata Corp., College Station, Texas, USA). *P* values less than 0.05 were considered statistically significant.

## Results

**Fig. 2:**
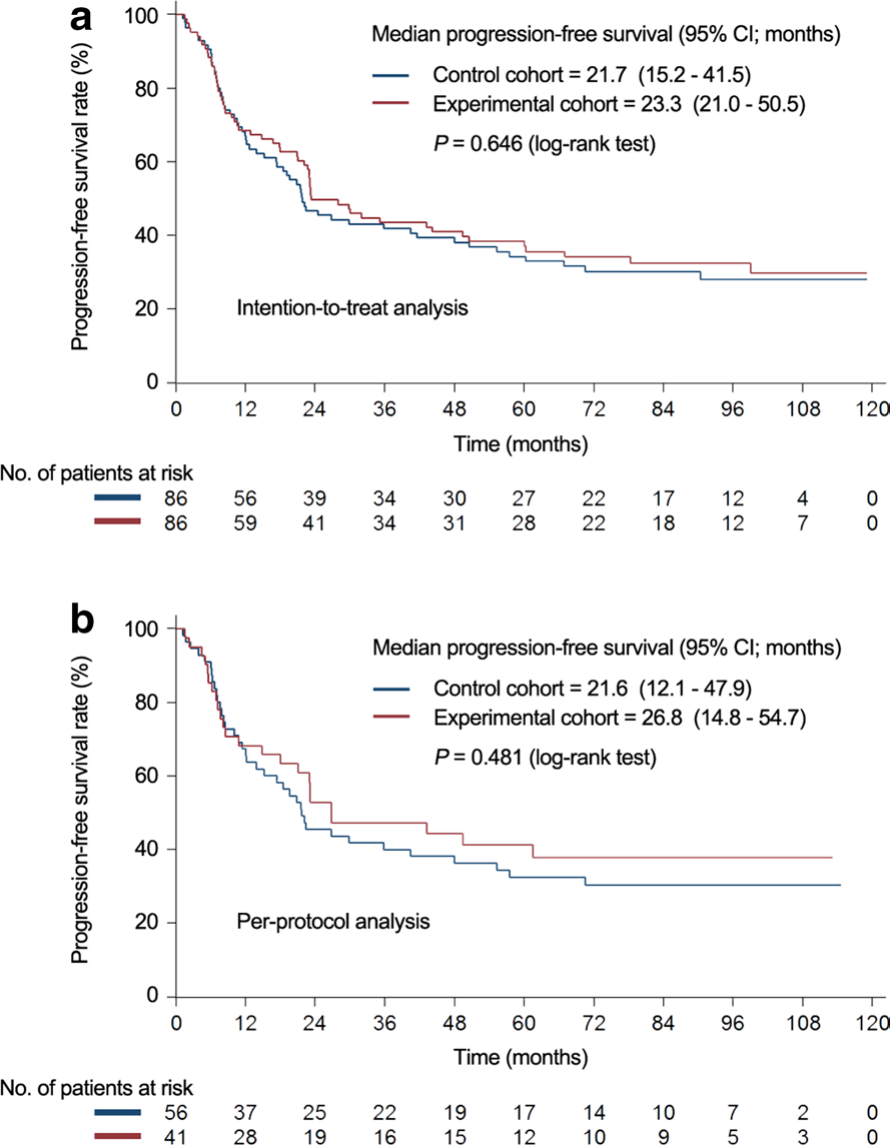
Comparison of progression-free survival rates by intention-to-treat (**a**) or per-protocol (**b**) analyses. *CI* confidence interval

**Fig. 3:**
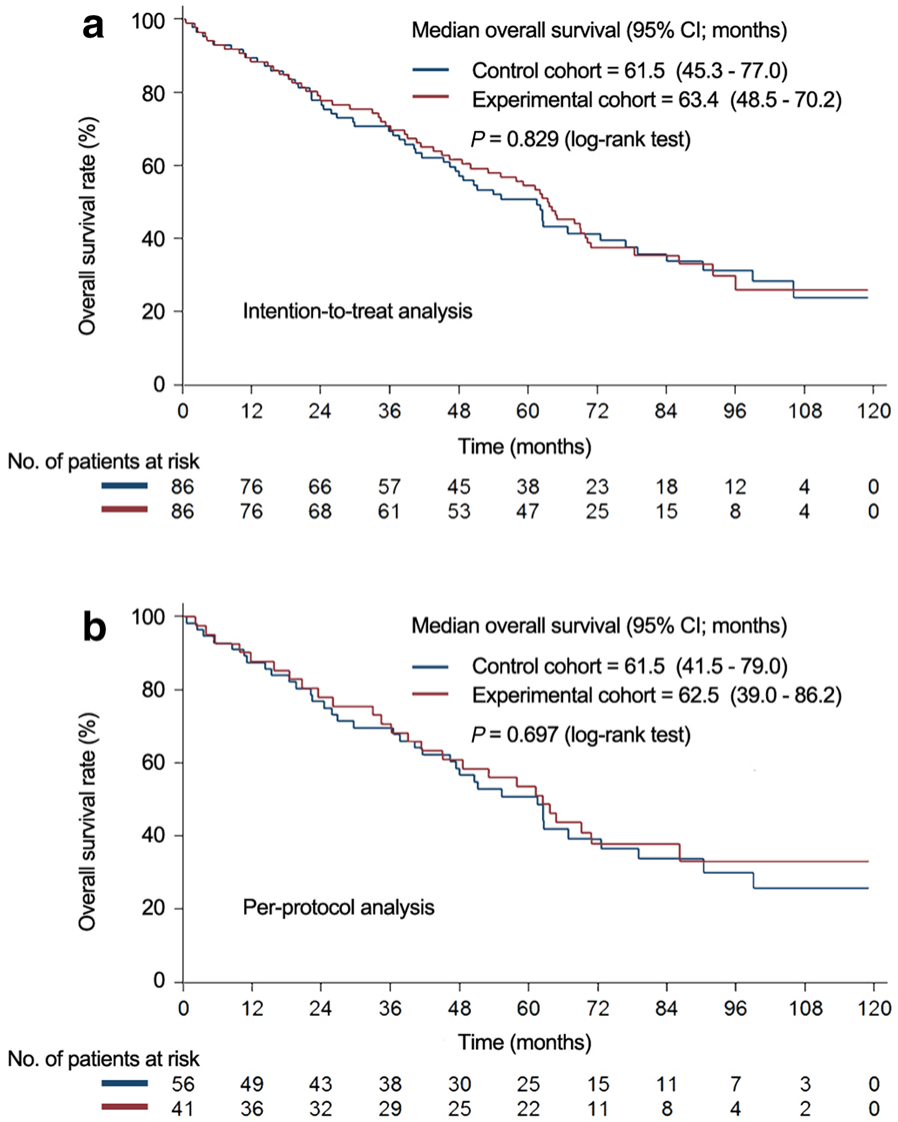
Comparison of overall survival by intention-to-treat (**a**) or per-protocol (**b**) analyses. *CI* confidence interval

The Kaplan–Meier progression-free survival and overall survival curves are shown in Fig. 2, with both intention-to-treat and per-protocol analyses. The median progression-free survival showed no difference between the control cohort and the experimental cohort (intention-to-treat analysis: 21.7 vs. 23.3 months, *P* = 0.646; per-protocol analysis: 21.6 vs. 26.8 months, *P* = 0.481). In addition, the median overall survival showed no significant difference between these two cohorts (intention-to-treat analysis: 61.5 vs. 63.4 months, *P* = 0.829; per-protocol analysis: 61.5 vs. 62.5 months, *P* = 0.697) (Fig. 3).

## Discussion

Whether based on intention-to-treat analysis or per-protocol analysis, we found no significant difference with respect to progression-free survival or overall survival between the control cohort and the experimental cohort. Our findings can be echoed by a detailed examination of the original GOG 114 and GOG 172 trials. In terms of progression-free survival, the advantage of median survival (intraperitoneal treatment group vs. intravenous treatment group) was not significantly different for the GOG 114 and GOG 172 trials (6.0 months [27.9 vs. 22.2 months] for the GOG 114 trial; 5.5 months [23.8 vs. 18.3 months] for the GOG 172 trial). The overall survival was longer in the GOG 172 trial (11.0 months [63.2 vs. 52.2 months] for the GOG 114 trial; 15.9 months [65.6 vs. 49.7 months] for the GOG 172 trial). However, in the GOG 172 trial, patients were recruited between March 1998 and January 2001; during this time, two drugs were available for second-line treatment: topotecan, which was approved in May 1996, and liposomal doxorubicin, which received accelerated approval in June 1999. These drugs may potentially extend post-progression survival and, thus, overall survival [8].

In summary, this propensity score-matching study of patients with stage III epithelial, tubal, and peritoneal cancers showed that patients in both the control cohort and the experimental cohort had similar survival outcomes, suggesting that the addition of a second intraperitoneal paclitaxel dose (administered every 3 weeks) to the current standard platinum-based intraperitoneal chemotherapy regimen does not yield significant incremental survival benefits. If our findings could be confirmed by a prospective randomized study, then it would be interesting to explore the efficacy of shifting back to a dose-dense intraperitoneal delivery of paclitaxel or a dose-dense delivery of a new formulation of paclitaxel for the patients with stage III epithelial ovarian, tubal, and peritoneal cancers.
